# Analyzing large-scale conservation interventions with Bayesian hierarchical models: a case study of supplementing threatened Pacific salmon

**DOI:** 10.1002/ece3.1509

**Published:** 2015-04-27

**Authors:** Mark D Scheuerell, Eric R Buhle, Brice X Semmens, Michael J Ford, Tom Cooney, Richard W Carmichael

**Affiliations:** 1 Fish Ecology Division, Northwest Fisheries Science Center, National Marine Fisheries Service, National Oceanic and Atmospheric AdministrationSeattle, Washington, 98112; 2 Scripps Institute of Oceanography, University of CaliforniaSan Diego, La Jolla, California, 92093; 3 Conservation Biology Division, Northwest Fisheries Science Center, National Marine Fisheries Service, National Oceanic and Atmospheric AdministrationSeattle, Washington, 98112; 4 Northeast-Central Oregon Research and Monitoring, Oregon Department of Fish and Wildlife, Eastern Oregon UniversityLa Grande, Oregon, 97850

**Keywords:** Before–after control–impact, captive breeding, hatchery, multivariate, salmon, supplementation, time series

## Abstract

Myriad human activities increasingly threaten the existence of many species. A variety of conservation interventions such as habitat restoration, protected areas, and captive breeding have been used to prevent extinctions. Evaluating the effectiveness of these interventions requires appropriate statistical methods, given the quantity and quality of available data. Historically, analysis of variance has been used with some form of predetermined before-after control-impact design to estimate the effects of large-scale experiments or conservation interventions. However, ad hoc retrospective study designs or the presence of random effects at multiple scales may preclude the use of these tools. We evaluated the effects of a large-scale supplementation program on the density of adult Chinook salmon *Oncorhynchus tshawytscha* from the Snake River basin in the northwestern United States currently listed under the U.S. Endangered Species Act. We analyzed 43 years of data from 22 populations, accounting for random effects across time and space using a form of Bayesian hierarchical time-series model common in analyses of financial markets. We found that varying degrees of supplementation over a period of 25 years increased the density of natural-origin adults, on average, by 0–8% relative to nonsupplementation years. Thirty-nine of the 43 year effects were at least two times larger in magnitude than the mean supplementation effect, suggesting common environmental variables play a more important role in driving interannual variability in adult density. Additional residual variation in density varied considerably across the region, but there was no systematic difference between supplemented and reference populations. Our results demonstrate the power of hierarchical Bayesian models to detect the diffuse effects of management interventions and to quantitatively describe the variability of intervention success. Nevertheless, our study could not address whether ecological factors (e.g., competition) were more important than genetic considerations (e.g., inbreeding depression) in determining the response to supplementation.

## Introduction

Human activities such as habitat modification, alteration of biogeochemical cycles, overharvest, and spread of non-native species affect all of the earth's ecosystems (Vitousek et al. 1997), increasing extinctions of both terrestrial (Hoekstra et al. 2005) and marine species (Dulvy et al. 2003). In response, a variety of conservation actions have been employed to recover or prevent the extinction of at-risk species. Habitat restoration efforts in both terrestrial and aquatic ecosystems are now widespread (van Andel and Aronson 2012), but their effects can be limited. For example, reforested plantations (Chazdon 2008) and organic farms (Gabriel et al. 2010) have enhanced local biodiversity, but they have not matched the composition and structure of the original landscapes they replaced. Protected reserves are used increasingly in marine (Mora et al. 2006) and terrestrial ecosystems (Jenkins and Joppa 2009), but measures of their effectiveness vary broadly due to mobility of animals, poaching, data quality, and interpretation of effects (Kaplan et al. 2013). Captive breeding programs have offered hope for animals facing imminent extinction, but high costs and negative genetic impacts can limit their application (Williams and Hoffman 2009).

In most rivers along the west coast of the continental United States, populations of *Oncorhynchus* spp. (Pacific salmon) have been reduced to small fractions of their historical abundances and are the focus of widespread conservation efforts. For these purposes, Pacific salmon species are grouped into evolutionarily significant units (ESUs), defined as a group of salmon that (1) is reproductively isolated from other conspecific populations, and (2) represents an important component in the evolutionary legacy of the species (Waples 1991). Currently, 28 of the 49 extant Pacific salmon ESUs are listed as “threatened” or “endangered” under the US Endangered Species Act (ESA). A wide variety of anthropogenic causes (e.g., habitat loss, hydropower development, overharvest) and natural drivers (e.g., climate variability) have contributed to these declines (Ford 2011).

Efforts to rebuild depressed populations are extensive and expensive. For example, in the Columbia River Basin, which contains 13 listed ESUs of Pacific salmon, more than 15,000 habitat restoration projects have been undertaken at an annual cost of over $150 million USD (Barnas and Katz 2010). In addition, artificial propagation of salmon has been used widely as a mitigation measure for more than a century. In the US Pacific Northwest, salmon hatcheries release about 400 million juveniles per year at a cost of roughly $40 million USD (Naish et al. 2008). Many of these fish are produced to meet tribal, commercial, or recreational harvest demands, or to mitigate for habitat loss. However, since the mid-1980s, hatcheries have been used increasingly to rebuild wild populations through supplementation programs, in which hatchery fish are encouraged to return to spawn in natural streams (Waples et al. 2007). Despite their widespread use, however, the effectiveness of these programs in achieving conservation goals remains poorly understood (Waples et al. 2007; Neff et al. 2011).

When designed appropriately a priori, large-scale interventions can be treated as large-scale experiments, with effect sizes estimated through carefully constructed analysis of variance (ANOVA) applied to data from before–after control–impact (BACI) studies (e.g., Keough and Quinn 2000). However, we often seek to estimate effect sizes following a natural disturbance or “unplanned experiment” (e.g., Buhle et al. 2009), when it is impractical or simply too late to assign experimental units randomly; in such cases, no true “control” exists (Stewart-Oaten and Bence 2001). Additional problems can arise when model assumptions are violated with respect to homogeneity of variance and uncorrelated errors (Carpenter et al. 1989; Underwood 1994).

Time-series models overcome these limitations by addressing explicitly the sequential nature of monitoring data. In particular, hierarchical or “state-space” models have two components that make them amenable to observational ecological studies (Royle and Dorazio 2008) that lack an explicit experimental design: (1) a process component, which describes the underlying dynamics of a true but unobserved state, and (2) an observation component, which relates the state(s) to an associated series of observations (the data). In addition, hierarchical models can accommodate missing data, different error distributions, and data from varying sources (e.g., visual surveys and net samples). Hierarchical models have a long history in fields such as engineering and economics (West and Harrison 1997), and reports of their application are now increasingly common in the ecological literature, especially in meta-analyses that examine effects across multiple spatial or temporal scales (e.g., Bennett and Adams 2004; Kulmatiski et al. 2008; Gabriel et al. 2010).

**Figure 1:**
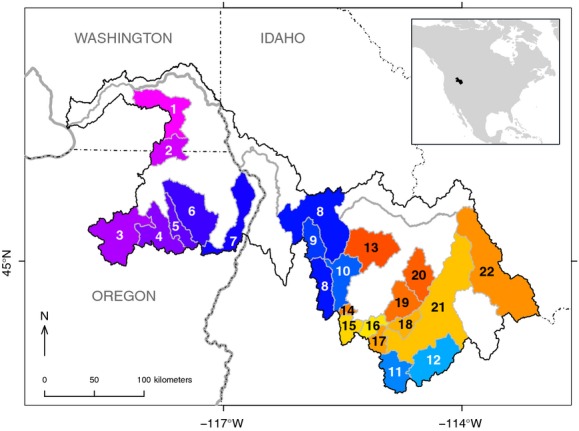
Map of the Snake River spring/summer Chinook salmon ESU (black outline) showing the supplemented populations (numbers 1–12 in purple/blue colors) and reference populations (numbers 13–22 in yellow/orange colors) used in the analysis (1: Tucannon R; 2: Wenaha R.; 3: Grand Ronde R. – Upper Mainstem; 4: Catherine Cr.; 5: Minam R.; 6: Lostine R.; 7: Imnaha R.; 8: South Fork Salmon R. – Mainstem; 9: Secesh R.; 10: South Fork Salmon R. – East Fork; 11: Salmon R. – Upper Mainstem; 12: Salmon R. – East Fork; 13: Big Cr.; 14: Sulfur Cr.; 15: Bear Valley Cr.; 16: Marsh Cr.; 17: Valley Cr.; 18: Salmon R. – Yankee Fork; 19: Loon Cr.; 20: Camas Cr.; 21: Salmon R. – Lower Mainstem; 22: Lemhi R.). Inset map shows the location of the ESU within North America.

Here, we used a form of hierarchical time-series model that is used commonly for analyzing intervention effects in financial markets (e.g., effect of a promotional campaign on consumer spending; West and Harrison 1997) to examine the effects of large-scale hatchery supplementation on spring- and summer-run *O. tshawytscha* (Chinook salmon) from the Snake River basin, which encompasses regions of Washington, Oregon, and Idaho in the northwestern United States (Fig.1). The Snake River spring- and summer-run (SRSS) ESU is one of 16 *O. tshawytscha* ESUs and was listed under the ESA in 1992. Using 43 years of monitoring data, we asked whether 11–23 years of supplementation have increased the density of naturally produced adults (i.e., fish that were born in the wild, not reared in a hatchery) in 12 supplemented populations, and if so, by how much. We found that, on average, supplementation has increased adult density among the 12 supplemented populations by only 3.3%.

## Materials and Methods

### Study species and data

Adult *O. tshawytscha* spawn in rivers and streams in late summer, and their eggs are buried in a nest (redd), where they incubate over winter before emerging as juveniles in spring. Juveniles from populations within the SRSS ESU then rear in fresh water for approximately 1 year before migrating to sea during the spring of their 2nd year. After spending 1–4 years foraging in the northeast Pacific Ocean, mature adults return from the ocean and migrate upstream to spawn in their natal streams (i.e., returning adults are 3–6 years old; >85% are age 4 or 5).

Our data set included information from 12 supplemented and 10 unsupplemented reference populations (Fig.1), although some populations were not sampled in every year. In addition, data collection in the Tucannon River (a supplemented population) did not begin until brood year 1979. None of the missing data posed any problems for our analyses, however, because the hierarchical model described below imputes the true density for all populations and years, regardless of whether or not we have a direct estimate for a specific population or year. Furthermore, although populations from the Wenaha and Minam rivers were never intentionally supplemented, they did in fact receive some level of supplementation through straying of hatchery adults. Therefore, we included them as supplemented populations in our primary analysis, but then repeated the analysis after excluding them from the data set.

We used data on the numbers and age structure of spawning adults provided by the Interior Columbia Technical Recovery Team (Ford 2011). We divided numbers of fish by hectares of available spawning habitat to standardize experimental effects across populations from different sized watersheds. The estimated area of available spawning habitat for each population was based on wetted channel width derived from 200-m reaches within the current spawning distribution, as delineated in a GIS derived from the 1:100,000-scale National Hydrography Dataset (Ford 2011).

Abundance and productivity data for fishes are commonly indexed by “brood year,” or the year during which eggs were spawned. For example, the total number of adult Chinook salmon produced from brood year 2004 would be the sum of all 3-, 4-, 5-, and 6-year-old adults returning in calendar years 2007, 2008, 2009, and 2010, respectively. Thus, although adult survey data were complete through calendar year 2012, we necessarily restricted our analyses to brood years 1964–2006 to allow for a full accounting of the entire life cycle. Referencing the data by brood year also allowed us to easily track any subsequent intervention effects on the density of natural-origin adults in the years following supplementation, as discussed below.

### Hatchery supplementation

**Figure 2:**
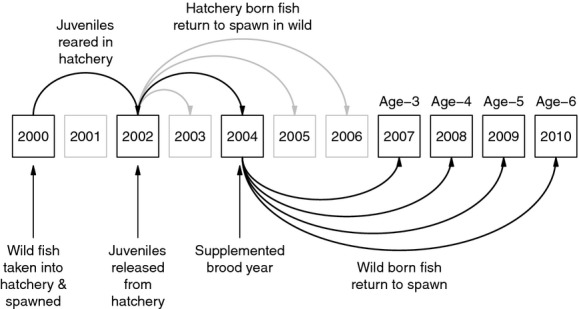
Diagram of the general model for supplementation evaluation. In this example, natural-origin adults are captured on the spawning grounds in 2000, brought into the hatchery, and spawned. Two years later, their offspring are released as smolts, which migrate to sea, and then return as adults over the following 1–4 years, such that brood years 2003–2006 are all then considered supplemented. For the 2004 brood, the total returning adults is then the sum of all 3-, 4-, 5-, and 6-year-old adults returning in 2007, 2008, 2008, and 2010, respectively. Note that sometimes hatcheries release juveniles after 1 year, but the same idea applies.

In general, hatchery supplementation programs try to select natural-origin adults for broodstock (Fig.2). Juveniles are then reared from the eggs in a relatively safe environment, which reduces the high mortality they would otherwise experience in the wild. Juveniles are then released back into rivers and streams, from which they ultimately migrate to sea, and to which they return to spawn as adults. A primary goal of supplementation programs is to increase the production of natural-origin adults. Thus, we were not simply interested in whether releasing more juveniles led to more returning adults of the same generation (i.e., whether hatchery-reared juveniles had greater survival from egg to adulthood). Rather, we sought to determine whether augmentation of the adult spawning population by hatchery-produced adults led to greater densities of natural-origin adults in the *following* generation. That is, a given population was considered supplemented in a brood year if fish born and reared in a hatchery were found on natural spawning grounds as adults (see Fig.2). Because we were interested in the overall effects of naturally spawning hatchery fish on subsequent natural-origin abundance, we considered a population to be supplemented if any adult hatchery-origin fish were present, regardless of whether they were intended to spawn there or had strayed from a neighboring hatchery.

**Figure 3:**
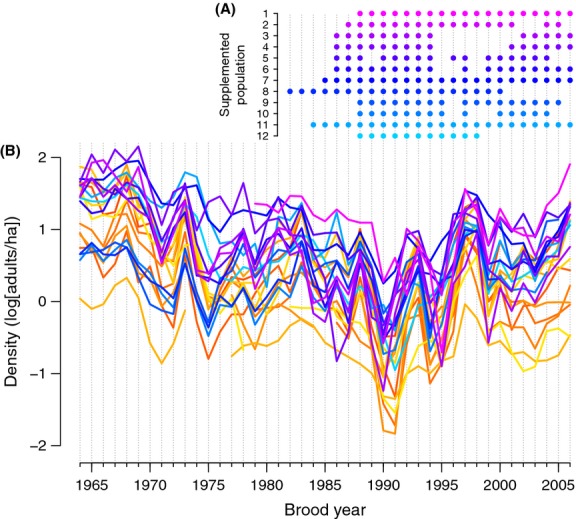
Time series of the supplemented years (A) and densities of adult Chinook salmon (B) indexed by brood year; colors are the same as in Figure1. Numbers on the *y*-axis in (A) refer to the 12 supplemented populations shown in Figure1; dots indicate populations and brood years in which the parents' generations were supplemented (see Methods for details). Breaks in some time series in (B) indicate missing years of data.

Hatchery supplementation in this region began in the early 1980s, but efforts were not uniform across time or the ESU (Fig.3A). Some populations (e.g., Tucannon R.) received continued supplementation, whereas others (e.g., Lostine R.) had alternating periods with supplementation turned on or off. Thus, for each population *i* in brood year *t*, we treat supplementation as a binary indicator variable *I*_*i,t*_ to indicate whether supplementation is “on” (*I*_*i,t*_ - 1) or “off” (*I*_*i,t*_ - 0). In our model described below, however, we require the actual shift, if any, in state *S*_*i,t*_ - *I*_*i,t*_ *– I*_*i,t*–1_ when supplementation is turned on (i.e., *S*_*i,t*_ - 1 – 0 - 1), turned off (i.e., *S*_*i,t*_ - 0 – 1 - −1), remains on (i.e., *S*_*i,t*_ - 1 – 1 - 0), or remains off (i.e., *S*_*i,t*_ - 0 – 0 - 0). For any reference population *i*, *I*_*i,t*_ - 0, and hence *S*_*i,t*_ - 0 – 0 - 0 for all *t*.

### Hierarchical time-series model

Census data on at-risk species are typically incomplete across time and space (i.e., lots of missing values) and characterized by relatively large sampling and observation errors (e.g., nonexhaustive counts, misidentification), which can confound parameter estimation and subsequent inference regarding population viability (Holmes 2001; Holmes and Fagan 2002). Thus, we used a multivariate, hierarchical time-series model to describe year-to-year changes in population density of natural-origin spawners. This approach offers a parsimonious, phenomenological description of population dynamics that allows us to estimate supplementation effects instead of focusing on the various functional forms of population dynamics.

We used a form of hierarchical time-series model that is common in financial analyses of promotional campaigns (West and Harrison 1997). In general, the model treats consumer demand for a product as a stochastic process that might include a trend, seasonal effects (e.g., sales of ice cream generally decrease in winter), or external influences (e.g., sales of bottled water increase during a heat wave). For example, a manufacturer may initiate a promotional campaign in an effort to increase sales of a product. Following the onset of advertising, the manufacturer uses the hierarchical time-series model to evaluate how much sales increased as a result of the promotion after accounting for other market forces.

In any given year, the spawning adults from any population are a mix of overlapping generations, so we modeled density as a biased random walk, such that

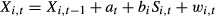
1

Here, *X*_*i,t*_ is the true but unobserved density (log-transformed adults ha^−1^) of natural-origin spawning adults from population *i* born in brood year *t*; *a*_*t*_ is an annual growth rate common to all populations (i.e., it reflects large-scale drivers of temporal variation); *b*_*i*_ is the effect of supplementation on population *i*; and *S*_*i,t*_ is the supplementation indicator described above for population *i* in brood year *t*. Finally, *w*_*i,t*_ is a random process error representing environmental stochasticity.

Specifically, we modeled annual population growth rate (*a*_*t*_) as a first-order Markov process because the large-scale drivers of environmental variability important to salmon survival (e.g., upwelling currents, temperature) tend to be highly autocorrelated from year to year (Zabel et al. 2006; Scheuerell et al. 2009). Thus,

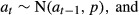
2a

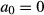
2b

We set the initial growth rate (*a*_0_) equal to zero because its estimation is confounded with the initial state (*X*_*i,*0_). We assigned the precision (i.e., the inverse of the variance 1/*p*) a Gamma(0.001, 0.001) prior.

We treated supplementation effects as random and drawn from a normal distribution with mean *m*_*b*_ and variance *c*. This allowed us to examine not only site-specific effects of supplementation, but also to evaluate the ESU level mean effect of supplementation. Thus, if population *i* is within the supplemented set, then

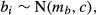
4and *b*_*i*_ - 0 if *i* is within the reference set. Following Gelman (2006), we assigned noninformative Unif(−100, 100) and Unif(0, 100) priors to the mean (*m*_*b*_) and standard deviation (*c*), respectively, of the random effects.

We used the estimates of *b*_*i*_ to calculate the percent change in population density owing to supplementation, which follows from equation 1. If the log-density in a nonsupplemented state for population *i* is *x*_*i*_, then the log-density in its supplemented state is *x*_*i*_ + *b*_*i*_. Therefore, the percent change in density is [exp(*x*_*i*_ + *b*_*i*_) – exp(*x*_*i*_)]/exp(*x*_*i*_), which reduces to simply exp(*b*_*i*_) – 1.

The variance of the process errors *w*_*i*,*t*_ differs among populations to reflect any residual heterogeneity in local environmental conditions not captured by the random year or supplementation effects, such that

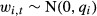
5

We assigned the process precision (i.e., the inverse of the process variance 1/*q*_*i*_) a Gamma(0.001, 0.001) prior. For each population, we assumed the initial state at *t *-* *0 (*X*_*i*,0_) was also random with an unknown mean (*m*_*X*0_) and a fixed and relatively uninformative variance of 10^4^, such that

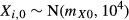
6

As mentioned above, the hierarchical framework further accommodates sampling or observation errors that may exist in our density measurements. Specifically, *Y*_*i,t*_ is the observed density of spawning adults (log-transformed adults ha^−1^) from population *i* born in year *t*, which is corrupted by a normally distributed observation error *v*_*i*,*t*_, such that

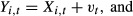
7

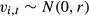
8

In this case, we assumed the observation variance *r* does not vary among populations because similar methods were used to enumerate spawning adults (see Appendix S1 in Supporting Information for alternative assumptions about variance structures). We assigned the precision of the observation errors (i.e., the inverse of the observation variance 1/*r*) a Gamma(0.001, 0.001) prior, which should be minimally informative given the large number of groups and time points in our analysis (Gelman 2006).

We used Bayesian inference to estimate all model parameters and the unobserved true state of annual natural spawner densities in each population. We used the freely available R v3.0.2 software (R Development Core Team 2013) combined with the JAGS v3.4.0 software (Plummer 2003) to perform Gibbs sampling with 10 parallel chains of 4 × 10^5^ iterations. Following a burn-in period of 6 × 10^5^ iterations, we thinned each chain by keeping every 400th sample to eliminate any possible autocorrelation, which resulted in 10^4^ samples from the posterior distributions. We assessed convergence and diagnostic statistics via the CODA package in R (Plummer et al. 2006). Specifically, we used visual inspection of trace plots and density plots and verified that Gelman and Rubin (1992) potential scale reduction factor (*R*_*hat*_) was less than 1.1, to ensure adequate chain mixing and parameter convergence (the maximum value of *R*_*hat*_ was 1.002 across all parameters and states). See Appendix S1 in Supporting Information for R and JAGS code.

We initially considered additional forms of hierarchical models that differed with respect to random or fixed effects of year and supplementation, as well as different variance–covariance structures (see Appendix S2 in Supporting Information). We used the deviance information criterion (DIC, Spiegelhalter et al. 2002) to evaluate relative support from the data for each of the competing models. Based on this initial model selection exercise, we present the structure and results only from the highest ranked model because the difference in DIC between first- and second-ranked models was extremely large (see Table S2 in Supporting Information).

## Results

Dramatic declines in densities of natural-origin adults across all 22 populations of Snake River spring/summer Chinook salmon were evident from the mid-1960s to the early 1990s, when the ESU was listed as threatened (Fig.3B). Supplemented populations then increased in natural spawner density into the late 1990s, as did reference populations. Following a peak in density around brood year 1997, both reference populations and treatment populations where supplementation had been stopped appeared to decrease in density more so than those populations that continued to receive hatchery supplementation. Prior to the onset of supplementation, populations that were ultimately chosen for supplementation appeared to have a higher mean density of natural spawners than reference populations.

We found very limited support for a supplementation effect at both the individual population and ESU levels (Table1). Mean values of the posterior distributions for the population-specific supplementation effects (*b*_*i*_) ranged from −0.00044 to 0.081, and the 95% credible intervals included 0 for all populations. Thus, on average supplemented populations increased by 0–8.4% relative to nonsupplemented years. The probability that *b*_*i*_ was positive (i.e., the intended direction) ranged from 0.50 to 0.84 for individual populations (Table1). Equivalently, then, there was a 16–50% chance that supplementation may have actually caused some decrease in densities of wild adults across the ESU. The hypermean of supplementation effects at the ESU level (*m*_*b*_) had a mean value of 0.033 and a 95% credible interval of −0.077 to 0.15; the probability that *m*_*b*_ was positive was 0.73 (Table1).

**Table 1:** Summary statistics for population-specific supplementation effects (*b*_*i*_) and their hypermean (*m*_*b*_), including the posterior mean, 95% credible interval (CI), and probability that *b*_*i*_ or *m*_*b*_ is positive

ID	Population	Mean	95% CI	Pr(+)
1	Tucannon R.	0.032	(−0.21, 0.27)	0.66
2	Wenaha R.	0.046	(−0.13, 0.29)	0.72
3	Grand Ronde R. – Upper Mainstem	0.025	(−0.16, 0.20)	0.63
4	Catherine Cr.	−0.00044	(−0.26, 0.16)	0.50
5	Minam R.	0.042	(−0.086, 0.17)	0.75
6	Lostine R.	0.0063	(−0.15, 0.13)	0.54
7	Imnaha R.	0.022	(−0.14, 0.17)	0.63
8	South Fork Salmon R. – Mainstem	0.081	(−0070, 0.36)	0.84
9	Secesh R.	0.025	(−0.19, 0.22)	0.63
10	South Fork Salmon R. – East Fork	0.068	(−0.070, 0.26)	0.83
11	Salmon R. – Upper Mainstem	0.0074	(−0.18, 0.15)	0.54
12	Salmon R. – East Fork	0.039	(−0.14, 0.25)	0.69
*m*_*b*_	Hypermean	0.033	(−0.077, 0.15)	0.73

When we repeated our analysis after excluding the Wenaha and Minam populations, which had some hatchery-origin adults but were never intentionally supplemented, the supplementation effect increased for all populations, but also tended to be more variable (Table S1). In this case, the supplemented populations increased by 1–13% relative to nonsupplemented years. In particular, the hypermean (*m*_*b*_) had a mean value of 0.056 and a 95% credible interval of −0.086 to 0.20; the probability that *m*_*b*_ was positive increased from 0.73 to 0.80.

**Figure 4:**
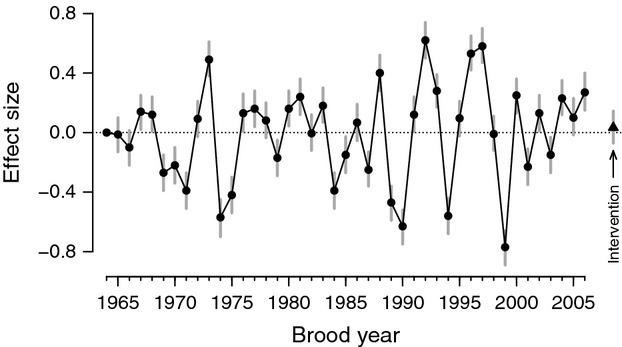
Time series of estimated year effects. Points are medians of the posterior distributions. Vertical bars indicate 95% credible limits for each year effect. For comparison, the median (triangle) and 95% credible limits for the mean of the experimental effects (*m*_*b*_) are also shown.

Year effects (*a*_*t*_), which accounted for large-scale temporal variation common to all populations across the ESU, were highly variable and generally much larger in magnitude than supplementation effects (Fig.4). Larger up-and-down swings in year effects appeared more commonly in the latter portion of the study period, particularly during the 1990s. The mean of the year effects was −0.041 during the first half of the time series when abundance declined across the entire ESU, but then jumped to 0.029 during the second half of the period as populations increased on average. Relative to the hypermean of supplementation effects, the magnitudes (absolute values) of the *a*_*t*_ were more than twice *m*_*b*_ for 39 of 43 years (Fig.4).

**Figure 5:**
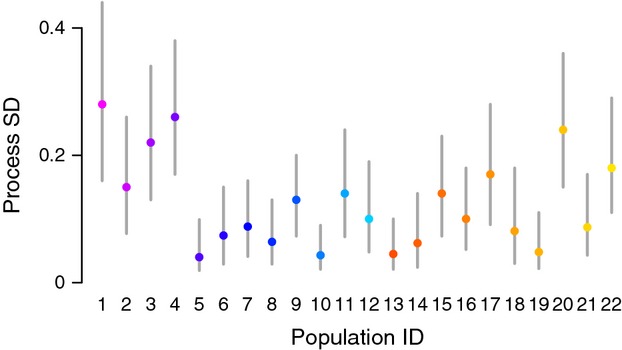
Estimated standard deviation (SD) of the process errors for each of the 22 populations. Colored points are medians of the posterior distributions. Gray vertical bars indicate 95% credible limits on the estimated SD. Colors and IDs are the same as in Figure1.

After controlling for supplementation and year effects, we found considerable variability among populations in the standard deviation of the process errors (Fig.5). In particular, populations from the western and eastern portions of the ESU had much larger variance in process residuals than those populations in the middle of the ESU. There was very little difference, however, in the average standard deviations of reference and supplemented populations (i.e., the mean of SD_*sup*_ – SD_*ref*_ was 0.016 with 95% credible limits of 0.0097 and 0.020).

## Discussion

We found that over varying timespans since the 1980s, hatchery supplementation of threatened *O. tshawytscha* has had rather minimal effects on increasing the density of naturally spawning adults. For example, in the East Fork Salmon River, we estimated with 95% probability that 11 consecutive years of supplementation (i.e., the fewest among all populations) ultimately produced somewhere between a 13% decrease and 28% increase in the density of natural-origin adults. Similarly, 23 successive years of supplementation in the Upper Mainstem Salmon River (i.e., the most among all populations) resulted in densities of natural-origin adults that were between 17% less and 16% greater than years prior to supplementation. Notably, the 95% credible interval of the estimated effect of supplementation spanned zero in all cases, indicating some nonzero probability that hatchery supplementation actually had negative impacts on natural-origin adults. Therefore, although that the probability of a positive effect of supplementation on spawning abundance was greater than 50% in all but one population, the effect appears small and uncertain compared to large-scale drivers of temporal variation (i.e., estimated year effects) such as climate, habitat alterations, and hydroelectric dam system operations.

There are a number of possible explanations for our failure to find strong evidence for a positive effect of supplementation. First, our findings are consistent with other studies, which indicate that hatchery-produced salmon often have poor reproductive success in the wild (Araki et al. 2008; Christie et al. 2014) and may even depress the abundance of wild adults (Buhle et al. 2009). Thus, although artificial propagation (including supplementation) may be a potentially useful intervention for preventing imminent extinction of specific populations (Neff et al. 2011), supplementation may be largely ineffective as a recovery tool for increasing the density of natural-origin adults within this ESU over the long term.

Second, the theoretical basis of supplementation assumes that target populations are well below carrying capacity (Cuenco 1994; Naish et al. 2008). However, whether this assumption is fulfilled is questionable in this ESU, and the failure of supplementation to increase abundance in our study may be that populations are closer to current carrying capacity than is generally appreciated. For example, a recent analysis of this same ESU of Chinook salmon found strong density-dependent survival of juveniles, despite reductions in spawning adults to orders of magnitude below historical numbers (Thorson et al. 2013). If habitat capacity has been reduced due to long-term structural alterations, then supplementation without concomitant habitat restoration will be unlikely to provide strong conservation benefits and may simply result in displacement of natural-origin fish by hatchery fish. Alternatively, if capacity reduction is due in part to losses of materials and energy provided by spawning and dead adult salmon (e.g., Scheuerell et al. 2005), then supplementation itself might be expected to help increase carrying capacity.

Finally, our study took a broad view of supplementation and considered the presence of any hatchery-origin fish in a population to be an indicator of supplementation. However, some of these fish were strays from hatchery programs using semidomesticated stocks never intended for supplementation, and it is possible that differences in hatchery practices may obscure a more positive signal from more recent programs using only “best practices” (e.g., Mobrand et al. 2005). Excluding the two populations that were never intentionally supplemented resulted in a larger but more variable estimate of the supplementation effect. Also, it is important to note that even if supplementation does result in a modest abundance increase, there are concerns that long-term use of artificial propagation could reduce genetic fitness (Araki et al. 2008), contribute to ecological risks such as competition for resources (Berejikian et al. 2000), and serve as vectors for diseases or parasites (Naish et al. 2008).

Massive efforts are underway worldwide to conserve at-risk species, and societies would like to know what they are getting for their investment. Our understanding of the efficacy of conservation interventions, or large-scale ecological experiments, depends on three important aspects. First, appropriate design considerations (e.g., replication, spacing, contrasts) are necessary to assess dynamic threats to biodiversity patterns and processes (Pressey et al. 2007). In particular, BACI designs, including paired and multiple BACI designs, are effective tools in evaluating both the effects of human development (e.g., Torres et al. 2011) and habitat improvements (e.g., Bro et al. 2004) on species of concern. For post hoc analyses such as the one illustrated here, however, we could not use a standard multiple BACI design, but we did use an approach that provided the necessary contrast in the model formulation, given the nonsystematic application of hatchery supplementation over very large spatial and temporal extents (i.e., our study spanned 56,764 km^2^ and 45 years), and missing data from some sites and years. Second, there is no substitute for adequate monitoring and data reporting (Downes et al. 2002; Bennett and Adams 2004). We were perhaps fortunate to study an ESA-listed species because widespread interest in recovery and conservation of these species encourages comprehensive reporting of monitoring data (Barnas and Katz 2010). Without such data, there can be no meaningful analysis of conservation efforts, regardless of their cost. Third, any inferences regarding the “significance,” size, and magnitude of experimental effect(s) will follow directly from the choice of statistical analysis (Osenberg et al. 1994; Carpenter et al. 1998; Downes et al. 2002). Here, we were specifically interested in estimating the hierarchical effects of supplementation on populations within a larger ESU, but there would have been no way to do that with an ANOVA model. Standard ANOVA models must also be modified to account for changes in variance as opposed to shifts in mean state (Underwood 1994), but the Bayesian hierarchical model (BHM) framework allowed us to easily examine a variety of assumptions about possible step changes and gradual changes in environmental process variances.

We believe BHMs have several advantages in a general ecological context, specifically in cases that do not fit the standard BACI design. As Clark (2005) notes, BHMs can describe complex relationships because they allow for stochasticity at multiple levels of spatial and temporal organization (e.g., individuals within populations), they can incorporate disparate sources of information (e.g., visual counts and net samples), and they can estimate large numbers of unobserved variables and parameters. In addition, they provide not only an estimate of the central tendency, but also an explicit accounting and propagation of all sources of uncertainty throughout the entire model. Similar hierarchical approaches have become increasingly popular in ecological meta-analyses (e.g., Bennett and Adams 2004; Kulmatiski et al. 2008) and analyses of management effects on habitat occupancy and species diversity (e.g., Zipkin et al. 2010; Giovanini et al. 2013; Iknayan et al. 2014). Bayesian hierarchical models also allow for direct quantification of the probability that a parameter takes a specific value. In our case, we could state explicitly the probability that supplementation had a positive effect at both the population and ESU levels.

Ecologists have worked for decades to understand how natural disturbances and human impacts affect communities and ecosystems. In cases where highly replicated, randomized, and relatively small experimental units have been used, a simple statistical analysis can demonstrate whether the manipulations caused the observed effect (Carpenter et al. 1989; Downes et al. 2002). However, scaling experiments up to levels where conservation and management decisions must be made can yield invaluable insights that might otherwise remain obscured (see review by Carpenter et al. 1995). Such comprehensive evaluations require additional consideration as to how the data are analyzed. Ad hoc and unbalanced designs, the desire to incorporate random effects across multiple levels of organization, and correlations across time and space can all create problems for traditional approaches. Here, we have shown how Bayesian hierarchical models, which have been used effectively in other disciplines, can address these potential shortcomings and integrate information from a variety of sources to answer questions about ecological responses to a large-scale conservation intervention.

## Data Accessibility

The R and JAGS script for running the model is included in supplementary material (Appendix S1). All data used herein have been archived on a US government server and can be accessed at https://www.webapps.nwfsc.noaa.gov/apex/f?p-261:home:0

## Supporting Information

**Appendix S1.** R and JAGS script for fitting hierarchical intervention-effects model. **Appendix S2.** Alternative model formulations for estimating supplementation effects. **Table S1.** Summary statistics for population-specific supplementation effects (*b*_*i*_) and their hyper-mean (*m*_*b*_), including the posterior mean, 95% credible interval (CI), and probability that *b*_*i*_ or *m*_*b*_ is positive. These results pertain to the data set that excludes the Wenaha and Minam populations, which were never intentionally supplemented, but did receive some hatchery-origin strays from nearby populations. **Table S2.** Model selection results for alternative model formulations discussed in Appendix S2. The various model forms focused on changes to the year effect (**a**) and the variance-covariance matrices for the process (**Q**) and observation (**R**) errors.
