# Supplementary material for: Analyzing large-scale conservation interventions with Bayesian hierarchical models: a case study of supplementing threatened Pacific salmon
Source: Ecol Evol. 2015 Apr 27;5(10):2115–25. doi: 10.1002/ece3.1509 (PMC4449763; doi:10.1002/ece3.1509)
Supplement: Supplementary file 1 [file ece30005-2115-sd1.docx]

**SUPPORTING INFORMATION for ECE-2014-08-0384.R1**

**Appendix S1** R and JAGS script for fitting hierarchical intervention-effects model.

#-------------

# user inputs

#-------------

# file where to save JAGS model

file.JAGS <- "interventionModel.txt"

# number of reference popns

n.ref <- 10

# number of reference popns

n.int <- 12

#-------

# inits

#-------

# load necessary pkgs

library(runjags)

library(R2jags)

#----------

# get data

#----------

# data file is matrix with rows=n.yrs & cols=(n.ref+n.int)

# Y <- cbind(refData, intData)

# indicator file is matrix containing -1/0/1; dim(chi)==dim(Y)

# chi <- ...

# number of years of data

n.yrs <- nrow(Y)

#------------

# JAGS setup

#------------

# begin JAGS model description

cat("

# model specification

# i = popn

# t = brood year

# X = unobserved true log[spawner density] (ie, state)

# alpha = random effect of popn growth

# beta = random effect of supplmentation

# chi = indicator function (-1/0/1)

# Y = observations (log[spawner density])

# Q = process variance

# R = observation variance

# State eqn

# X_{i,t} = X_{i,t-1} + alpha_t + beta_i*chi_{i,t} + v_{i,t}

# v_{i,t} ~ N(0,Q_i)

# Obs eqn

# Y_{i,t} = X_{i,t} + w_{i,t}

# w_{i,t} ~ N(0,R)

data {

# calc total number of sites

n.tot <- n.ref + n.int

} # end data specification

model {

#--------

# PRIORS

#--------

# mean of initial states

X0.mu ~ dunif(-100,100);

# SD of initial states

X0.sig <- 100;

# precision of initial states

X0.tau <- 1/(X0.sig*X0.sig);

# priors for initial states

for(i in 1:n.tot) { X0[i] ~ dnorm(X0.mu,X0.tau) }

# alpha = random effect of year

# initial value is zero

alpha0 <- 0;

# var in RW for alpha

tau.Qa ~ dgamma(0.001,0.001);

sig.Qa <- 1/sqrt(tau.Qa)

# beta = random effect of supplementation (ie, beta = 0 for ref popns)

# hyper mean across all popns

beta.mu ~ dunif(-100,100);

# hyper SD across all popns

beta.sig ~ dunif(0,100);

# precision across all popns

beta.tau <- 1/(beta.sig*beta.sig);

# set priors for betas

for(i in 1:n.int) { beta[i] ~ dnorm(beta.mu,beta.tau) }

# Q = process variance

# assume different among all popns with no cov

# diffuse gamma prior on precision

for(i in 1:n.tot) {

tau.Q[i] ~ dgamma(0.001,0.001);

sigma.Q[i] <- 1/sqrt(tau.Q[i]);

}

# R = obs variance

# assume same among all popns with no cov

# diffuse gamma prior on precision

tau.R ~ dgamma(0.001,0.001);

sigma.R <- 1/sqrt(tau.R);

#------------

# LIKELIHOOD

#------------

# first year; no effect of supplementation at start of ts

# predicted bias

alpha[1] ~ dnorm(alpha0, tau.Qa);

for(i in 1:n.tot) {

X.mu[1,i] <- X0[i] + alpha[1];

# predicted level vector

X[1,i] ~ dnorm(X.mu[1,i], tau.Q[i]);

# evaluate likelihood for first year

Y[1,i] ~ dnorm(X[1,i], tau.R)

}

# years 2:T

for(t in 2:n.yrs) {

# predicted bias

alpha[t] ~ dnorm(alpha[t-1], tau.Qa);

# loop over popns

for(i in 1:n.ref) {

# mean of the state

X.mu[t,i] <- X[t-1,i] + alpha[t];

# estimated state

X[t,i] ~ dnorm(X.mu[t,i], tau.Q[i]);

# evaluate likelihood

Y[t,i] ~ dnorm(X[t,i], tau.R);

} # end loop over ref popns

for(i in (n.ref+1):n.tot) {

# mean of the state

X.mu[t,i] <- X[t-1,i] + alpha[t] + chi[t,i]*beta[i-n.ref];

# estimated state

X[t,i] ~ dnorm(X.mu[t,i], tau.Q[i]);

# evaluate likelihood

Y[t,i] ~ dnorm(X[t,i], tau.R);

} # end loop over sup popns

} # end t loop over year

} # end model description

", file=file.JAGS)

# end JAGS model description

# data to pass to JAGS

data.JAGS <- c("chi", "Y", "n.ref", "n.int", "n.yrs")

# params/states for JAGS to return

par.JAGS <- c("alpha", "beta", "beta.mu", "X",

"sigma.Q","sig.Qa","sigma.R")

# MCMC parameters

mcmc.length <- as.integer(1e6)

mcmc.burn <- as.integer(6e5)

mcmc.thin <- 400

mcmc.chains <- 10

mod.JAGS <- list(data=data.JAGS,

inits=NULL,

parameters.to.save=par.JAGS,

model.file=file.JAGS,

n.chains=mcmc.chains,

n.burnin=mcmc.burn,

n.thin=mcmc.thin,

n.iter=mcmc.length,

DIC=TRUE)

# start timer

timer.start <- proc.time()

# fit the model in JAGS & store results

mod.fit <- do.call(jags.parallel, mod.JAGS)

# stop timer

(run.time.in.min <- round(((proc.time()-timer.start)/60)["elapsed"], 0))

# save workspace

sav.JAGS <- paste("JAGSinterModel",Sys.Date(),

"iter",mcmc.length,

"burn",mcmc.burn,

"thin",mcmc.thin,

"nc",mcmc.chains,

sep="_")

save(list=ls(), file=paste(sav.JAGS,"RData",sep="."))

#------------------

# JAGS diagnostics

#------------------

# summary of JAGS output

print(mod.fit)

# summary plots of JAGS output

plot(mod.fit)

# Gelman diagnostics

# need to unpack separate chains

mcmcList <- vector("list",length=dim(mod.fit$BUGSoutput$sims.array)[2])

for(i in 1:length(mcmcList)) {

mcmcList[[i]] <- as.mcmc(mod.fit$BUGSoutput$sims.array[,i,])

}

mcmcList <- mcmc.list(mcmcList)

# table of Rhat (pt est, upper CI)

(gmDiag <- gelman.diag(mcmcList))

# maximum Rhat across all params/states

gmDiag$psrf[which(gmDiag$psrf[,1]==max(gmDiag$psrf[,1])),]

# plots of Rhat over iteration

for(i in 1:nvar(mcmcList)) { gelman.plot(mcmcList[,i]) }

**Appendix S2** Alternative model formulations for estimating supplementation effects.

In addition to the hierarchical model presented in the main body of the manuscript, we also tried several alternative formulations to estimate supplementation effects, which we outline here. To begin, we write out the general model in matrix notation, such that the process model becomes

**X***_t_* = **X***_t_*_-1_ + **a***_t_* + **BS***_t_* + **w***_t_*.

In this case, **X***_t_* is an *i* x 1 vector of unknown states (*i* is the number of populations) at time *t*, **a***_t_* is an *i* x 1 vector of year effects, **B** is an *i* x *i* diagonal matrix of supplementation effects, **S***_t_* is an *i* x 1 vector of 1s and 0s indicating whether or not population *i* was supplemented in year *t*, and **w***_t_* is an *i* x 1 vector of process errors, such that

**w***_t_* ~ MVN(**0**,**Q**),

and **Q** is a variance-covariance matrix for the process errors. The observation model is simply

**Y***_t_* = **X***_t_* + **v***_t_*,

where **Y***_t_* is an *i* x 1 vector of observed spawner densities, and **v***_t_* is an *i* x 1 vector of observation (sampling) errors, such that

**v***_t_* ~ MVN(**0**,**R**),

and **R** is a variance-covariance matrix for the observation errors.

One alternative we considered is that the elements of **a***_t_* follow a purely random process, such that

**a***_t_* ~ MVN(**m***_a_*,**C***_a_*).

Because the year effects are shared among all populations (i.e., all elements of **a***_t_* are the same), the matrix form reduces to the univariate case. Another possible alternative would be to assume that the year effects (**a***_t_*) are instead fixed rather than random, but that would mean estimating as many year effects as there are years (i.e., 39 in our case), and may not provide much meaningful information anyway.

Other cases to consider include assumptions about the form of the process and observation variance-covariance matrices, **Q** and **R**, respectively. One can decide *a priori* whether or not any of the variance terms should be shared among populations. Similarly, one can decide whether or not to estimate any covariance(s), and if so, whether or not any of the covariance terms should be shared among populations.

In our case, we initially allowed for possible site-specific differences in the variance of the observation errors, but we had good reason to believe it could be shared given similarities in sampling programs and the manner in which data were collected. Thus, we compared the following forms for R (ignoring covariances for moment):

or .

From a model-fitting standpoint, the form on the left has *i* parameters whereas the more simple form on the right has only 1, and for which we ultimately found overwhelming support from the data. In an analogous manner, one could evaluate different hypotheses about the covariance structure, such that (ignoring possible differences in variances) one might compare

to .

We chose to model all populations as following their own process rather than treat them as multiple observations of a single process. That is, it seemed most reasonable that each population should reflect its own unique dynamics. Additionally, our random year effect accounted for large-scale temporal covariance, and therefore we set all covariance elements in both **Q** and **R** to 0. Those cases where we did, in fact, try to estimate covariance parameters had either very high DIC values compared to other models or they simply failed to converge for those parameters.

Of particular interest to us was whether there was any evidence from the data for a temporal change in the variance-covariance matrix **Q** for the process errors. To do so, we allowed for changes in **Q** under 2 scenarios: a step change and a linear trend. To begin, we defined **Q***_t_* to be a diagonal matrix with the time-dependent process variance of population *i* along the diagonal and zeroes elsewhere, such that

.

Next, examine evidence for a step-change *h* in the variance of those populations receiving supplementation, we set

*q_i_*_,_*_t_* = *q_i_* + *hS_i_*_,_*_t_* ,

and *S_i,t_* is a binary variable indicating whether (*S* = 1) or not (*S* = 0) supplementation affects population *i* in year *t* (*S* = 0 for all *t* in reference populations). To look for evidence of a linear change *k* in a variance over time, we set

*q_i_*_,_*_t_* = *q_i_* + *k*(*t* – *d_i_* + 1)*S_i_*_,_*_t_* ,

with *d_i_* as the first year of supplementation for population *i*. However, we found essentially no data support for this model and therefore used the more simple form presented in the main text.

**Table S1.** Summary statistics for population-specific supplementation effects (*b_i_*) and their hyper-mean (*m_b_*), including the posterior mean, 95% credible interval (CI), and probability that *b_i_* or *m_b_* is positive. These results pertain to the data set that excludes the Wenaha and Minam populations, which were never intentionally supplemented, but did receive some hatchery-origin strays from nearby populations.

ID Population Mean 95% CI Pr(+)

1 Tucannon R. 0.057 (-0.25, 0.37) 0.71

2 Wenaha R. NA NA NA

3 Grand Ronde R. – Upper Mainstem 0.068 (-0.17, 0.38) 0.76

4 Catherine Cr. 0.012 (-0.31, 0.19) 0.55

5 Minam R. NA NA NA

6 Lostine R. 0.066 (-0.089, 0.24) 0.80

7 Imnaha R. 0.042 (-0.14, 0.20) 0.70

8 South Fork Salmon R. – Mainstem 0.12 (-0.059, 0.41) 0.90

9 Secesh R. 0.040 (-0.23, 0.25) 0.67

10 South Fork Salmon R. – East Fork 0.089 (-0.065, 0.29) 0.87

11 Salmon R. – Upper Mainstem 0.015 (-0.20, 0.18) 0.57

12 Salmon R. – East Fork 0.060 (-0.15, 0.30) 0.74

*m_b_* hyper-mean 0.056 (-0.086, 0.20) 0.80

**Table S2.** Model selection results for alternative model formulations discussed in Appendix S2. The various model forms focused on changes to the year effect (**a**) and the variance-covariance matrices for the process (**Q**) and observation (**R**) errors. The options for **a** were Markov (as in the main text) or random (as in Appendix S2). In all cases the process errors were assumed to be independent (i.e., no covariance in **Q**), with the additional assumption that their variances were time invariant (as in the main text), followed a step-change with supplementation, or increased linearly during the period of supplementation. The observation errors were assumed to be independent and identically distributed (IID; as in the main text), independent but distributed differently (IDD), or non-independent and identically distributed (NID).

Rank **a Q R** ΔDIC

1 Markov Invariant IID 0

2 Random Invariant IID 154

3 Markov Invariant IDD 210

4 Markov Invariant NID 441

5 Random Invariant NID 451

6 Random Invariant IDD 551

7 Markov Linear IID 791

8 Markov Linear NID 859

9 Random Linear IDD 963

10 Random Linear NID 966

11 Markov Linear IDD 1346

12 Random Step IID 1441

13 Random Linear IID 1450

14 Markov Step IID 1520

15 Random Step IDD 1695

16 Markov Step NID 1776

17 Markov Step IDD 1903

18 Random Step NID 1946
